# Maternal inherited thrombophilia and recurrent pregnancy loss: a Tunisian study and review of literature

**DOI:** 10.4314/ahs.v23i4.52

**Published:** 2023-12

**Authors:** Rim Frikha, Fatma Turki, Nouha Abdelmoula, Tarek Rebai

**Affiliations:** 1 Department of Medical genetics; CHU Hedi Chaker, 3029 Tunisia; 2 University of Sfax, Faculty of Medicine of Sfax; Laboratory of histology, 3029 Tunisia

**Keywords:** Thrombophilia, recurrent pregnancy loss, factor V Leiden, prothrombin mutation

## Abstract

**Background:**

Inherited thrombophilia, mainly the Factor V Leiden (FVL) and Prothrombin mutation (PTM) are the most risk factors for venous thrombosis especially during pregnancy and was strongly associated with recurrent pregnancy loss (RPL), a devastating reproductive problem that affects more than 1% of couples who are trying to conceive. The frequencies also the correlation among these polymorphisms and RPL have been reported controversially in various populations.

**Objectives:**

In this study we evaluated the presence inherited thrombophilia amongst 35 Tunisian women with more than 2 miscarriages, referred to our genetic counseling.

**Methods:**

DNA was extracted from peripheral blood samples and PCR-RFLP was performed for the molecular diagnosis of mutation.

**Results:**

FVL and PTM were detected in 5.7 % and 2.9% respectively; in women with a particular history of early fetal loss and thrombotic events.

**Conclusion:**

This study emphasizes the importance of testing for FVL and FIIM in women with RPL; mainly in the context of thrombotic events. Multi-center collaboration is necessary to clarify the real impact of thrombotic molecular defects on the pregnancy outcome, to ascertain the effect of inherited thrombophilia on recurrent pregnancy loss and then to evaluate the appropriate therapeutic approach.

## Introduction

Pregnancy is a hypercoagulable state which causing a defective maternal hemostatic response and leading to thrombosis of the uteroplacental vasculature, that might cause pregnancy complications as recurrent pregnancy loss (RPL) 1. Recurrent pregnancy loss, defined by two or more failed pregnancies; is serious reproductive problem that affect more than 2% of couples who are trying to conceive 2. A variety of possible etiologies have been described. Often, inherited thrombophilia, mainly the Factor V Leiden (FVL) and Prothrombin G20210A mutation (PTM) are the most risk factors for venous thrombosis (VT) especially during pregnancy and was strongly associated with RPL 3,4.

The PTM (F2: 20210G > A (rs1799963)) results in increased Prothrombin levels. The FVL (F5 gene mutation 1691G > A (rs6025)) results in an altered variant of factor V, which enhance resistance to inactivation by protein C and hypercoagulable state with a five-to-tenfold risk of thrombosis in heterozygote and an 80-fold risk in homozygote individuals 5,6.

Many studies have investigated the relationship between both inherited thrombophilia and RPL, but the results were conflicting 7,8,,9,10.

There is convincing evidence that both mutations are associated with a double risk for unexplained RPL 11. As there are limited reports from Tunisia on the prevalence of inherited thrombophilia in reproductive failure, this study was carried out to determine the frequency and contribution of FVL and PTM in RPL. Interestingly, we are focused on clinical characteristics of carriers with history of RPL and thrombophilia, to delineate strategies for therapeutic prevention.

## Materials and methods

A total of 35 women with 2 or more miscarriages were enrolled in this study, carried out in Sfax, in South of Tunisia. In all the cases and informed consent and detailed reproductive histories were taken.

Genomic DNA was extracted from EDTA-anticoagulant blood samples and genotyping was performed by a duplex PCR-RFLP Assay for Simultaneous Detection of FV Leiden and Prothrombin G20210A Mutation as previously reported 12.

All statistical analysis was done using SPSS ver. 17 (statistical package for social sciences) software.

## Results

This study included 35 women with a history of 2 or more pregnancy losses. Clinical characteristics of couples with RPL are shown in Table 1. The range of age varied from 23 to 43 (with an average = 30 years). 31 women (88.6%) had primary RPL, and 4 women (11.4%) had secondary RPL. According to the number of miscarriages, the average was 3 pregnancy losses (2-6). 12 women had two pregnancy losses (34.3%) while 23 women experienced 3 or mores losses (65.7%).

**Table 1:** Clinical characteristics of women with RPL

Characteristics	Women with RPL
Age (median, years)	30 (23–42)
Miscarriages (median, range)	3 (2-6)
Women with two miscarriages (n, %)	12(34.3%)
Women with three or more miscarriages (n, %)	23(65.7%)
Women with primary RPL (n, %)	31 (88.6%)
Women with secondary RM (n, %)	4 (11.4%)
Week at which early miscarriages	7.6, +/-2.0
Occurred (mean +/- SD)	
Week at which late miscarriages	16.2, +/-1.4
Occurred (mean +/- SD)	
Women with Early RPL *(n, %)*	30 (82.9%)
Women with late RPL *(n, %)*	5 (8.6%)

RPL = recurrent pregnancy loss

30 RPL carriers have an early miscarriage which occurred at 7.6 weeks (SD +/-2.0). only 5 women (8.6%) experienced late miscarriages (mean= 16.2 weeks, SD +/-1.4). In 16 RPL carrier (45.7%) no associated alterations were found. While 19 women (54.3%) had at least one probable cause of RPL.

Among 35 carriers with RPL, thrombophilia was detected in three women with a particular history of embryonic loss (early RPL) and thrombotic events (8.6%). Factor V Leiden and Prothrombin mutation were identified in 5.7% and 2.9% respectively in the heterozygous model (GA1619 and GA20210).

## Discussion

This study was carried out to assess inherited thrombophilia in Tunisian Women with RPL. Overall, the frequency was 8.6%, in women with a particular history of embryonic loss and thrombotic events. Factor V Leiden and Prothrombin mutation were detected in 5.7% and 2.9% respectively.

**Figure 1:**
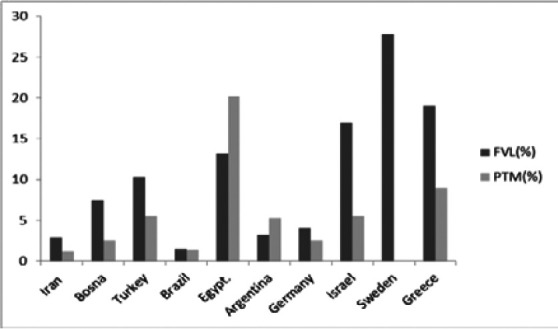
Worldwide frequency of FVL and PTM: review of literature

Initially, the prevalence of thrombophilia in our cohort is in the range of reported frequency in the literature –21 (Figure 1). Furthermore, the prevalence of each mutation (FVL or PTM) remains heterogeneous, due to several factors such as the sample size, in addition to geographic and ethnic variability worldwide 22-24. A high frequency of heterozygous genotypes (5-7%) in European people; while these heterozygotes are almost absent (<1.0%) in Asians and among African descendants 22-24.

Therefore, the heterogeneous rate of these mutations in various populations could affect the association between the FVL and PTM and RPL. In fact, the association between thrombophilia and RPL was largely investigated, and results were compiled in meta-analysis. 25,26 The FVL 1691G > A mutation and the risk of RPL confers a genetic contributing factor in increasing the risk of RPL 27. Women with early RPL had indeed a significantly increased carrier frequency of FVL mutation and the common OR being 1.68 (95% CI: 1.16-2.44) 28. Moreover; FVL carrier state may increase the susceptibility for early RPL 28. Regarding PTM, a recent meta-analysis suggests that the G20210A prothrombin mutation increases the risk of RPL (fetal loss, primary RPL, or secondary RPL), particularly in Europeans and women older than 29 years; with a combined odds ratio (OR) of 1.81 (95% confidence interval [CI]: 1.26-2.60) 29. Moreover; a positive relationship was significantly recorded between PTM and RPL during fetal loss, but not embryonic loss 29.

Despite the discrepancy of results, the pathophysiology of RPL related to the FVL or Prothrombin mutation is unique. Both thrombophilia led to placental tissue thrombosis, impair the placental blood supply, and activate the coagulation cascade as increased thromboxane production and annexin V displacement in the maternal blood circulation or at the feto-maternal interface. The risk of thrombosis in the presence of FII G20210A mutation was 2.4 in homozygous and heterozygous, higher than the normal population, While the risk of thrombosis with factor V Leiden is 2.66 times higher than that of the patients negative for this mutation (OR 2.66 95% CI 0.96-7.37 P=0.059); according to ACOG (American College of and Gynecologists Women's Health Care 2013). In addition; pregnant women with a family history of thrombosis present a 2.18-fold higher risk of thrombosis (OR 2.18 CI 0.9-5.26 P=0.085) 1.

While the results of our study demonstrated that genotyping of inherited thrombophilia are routine in most hospitals with genetic competence and that many similar studies are already published in the literature, and regardless of limited cases enrolled, these data highlights that testing for both mutations should be considered in women with RPL, especially in the context of thrombotic events. Thromboprophylaxis should be proposed for carrier of inherited thrombophilia and RPL to prevent fetal loss in subsequent pregnancy.

Nevertheless, the current guidelines agree that evidence is inadequate to recommend screening for FVL and PTM heterozygous as a low risk of thrombophilia, in women with RPL and thus do not recommend screening unless a personal history of venous thromboembolism is present 30 (Table 2). Moreover, a recent meta-analysis suggesting no benefit of LMWH in preventing recurrent pregnancy loss in women with inherited thrombophilia 31.

**Table 2:** The ACOG's treatment recommendations for FVL and PTM heterozygous

Clinical Scenario	Antepartum Management	Postpartum Management
without previous of VTE	Surveillance only prophylactic heparin	Surveillance (if no risk factors) anticoagulation (familial history of thrombotic episode)
with a single previous episode of VTE	Surveillance only prophylactic heparin	Postpartum anticoagulation therapeutic intermediate-dose heparin

Certain limitations should be considered when interpreting these results. Firstly, the limited sample size of our study. Then, the gestational age of pregnancy loss may also influence the frequency of inherited thrombophilia. A high prevalence of FVL in women with recurrent losses, especially in the second trimester of pregnancy; whereas; FII G20210A has been identified as a risk factor for recurrent loss in the first trimester 16. Finally; according to the number of miscarriages, previous study demonstrated that frequency is significantly higher in women with > 3 PL 32.

Finally, this study emphasizes the importance of testing for both mutations in women with RPL; mainly in the context of thrombotic events. Multi-center collaboration is necessary to clarify the real impact of thrombotic molecular defects on the pregnancy outcome, to ascertain the effect of thrombophilia on recurrent pregnancy loss and then to evaluate the appropriate therapeutic approach.
